# Assessment of bacterial and fungal (hemi)cellulose-degrading enzymes in saccharification of ammonia fibre expansion-pretreated *Arundo donax*

**DOI:** 10.1007/s00253-015-7066-3

**Published:** 2015-10-31

**Authors:** Simona Giacobbe, Venkatesh Balan, Salvatore Montella, Massimo Fagnano, Mauro Mori, Vincenza Faraco

**Affiliations:** Department of Chemical Sciences, University of Naples “Federico II”, Complesso Universitario Monte S. Angelo, via Cintia, Napoli, Italy; Department of Chemical Engineering and Materials Science, DOE Great Lakes Bioenergy Research Center, Michigan State University, Lansing, MI 48823 USA; Department of Agriculture, University of Naples “Federico II”, Portici, Napoli Italy

**Keywords:** Lignocellulose, Pretreatment, Cellulase, Arabinofuranosidase

## Abstract

This study reports enzymatic hydrolysis of the biomass of the giant reed (*Arundo donax* L.) after ammonia fibre expansion (AFEX) pretreatment. In particular, the capacity of the arabinofuranosidase from the fungus *Pleurotus ostreatus* recombinantly expressed in *Pichia pastoris* rPoAbf, its evolved mutant rPoAbf F435Y/Y446F and the endo-cellulase from *Streptomyces* sp. *G12* CelStrep recombinantly expressed in *Escherichia coli* to enhance the hydrolysis of AFEX-treated *A. donax* was investigated, using the corn stover as reference feedstock. The investigated enzymes were assayed using a mixture of purified cellulases (CBHI, CBHII, EGI and βG), endoxylanases (LX3, LX4) and accessory hemicellulases (LarbF and LβX) as reference enzyme mixture and substituting EGI with rCelStrep and LarbF with rPoAbf or rPoAbf F435Y/Y446F. The use of rPoAbf F435Y/Y446F in the substitution of LarbF led to improvements in sugar conversion, giving a glucan, xylan and arabinan conversion after 72 h of around 62, 63 and 80 %, respectively, similar or higher than those (44, 66 and 55 %) achieved by 72 h hydrolysis with commercial enzymes Novozymes Cellic®, Ctec3 and Htec3. The enzymes rPoAbf, rPoAbf F435Y/Y446F and rCelStrep were also investigated for their effect on hydrolysis of AFEX-pretreated *A. donax* by addition to commercial enzyme mixture Novozymes Cellic®, Ctec3 and Htec3, and it was shown that the addition of rPoAbf and its evolved mutant rPoAbf F435Y/Y446F enhanced both xylan and arabinan conversions, which achieved 80 % after 6 days of saccharification with rPoAbf F435Y/Y446F.

## Introduction

The policies for climate change mitigation promote the replacement of fossil fuels and petroleum-based products with alternative bioproducts from renewable resources such as biomass crops (Kajaste 2014). However, the incentives given to produce energy crops could have devastating effects on agricultural markets, as several food grain-producing lands will be taken away, reducing food production and consequently increasing food prices (Scheidel and Sorman 2012).

To avoid any competition for land between food and non-food crops, several efforts are under way to use crop lands not suitable for the traditional food crops for growing dedicated energy crops.

In Mediterranean environments, hilly areas are considered not appropriate for the traditional cereal production because yield and gross income are very low and because the traditional cropping system (deep soil tillage at the end of August and sowing in November) causes extreme vulnerability to soil erosion (Diodato et al. 2009, 2011; Fagnano et al. 2012). In these conditions, perennial biomass crops such as the giant reed (*Arundo donax* L.) proved to reduce soil erosion and to increase the potential gross income of farmers (Fagnano et al. 2015), with favourable environmental impacts (Forte et al. 2015).

The giant reed (*A. donax* L.) is also suggested for other areas not suitable for food crops such as polluted soils since it acts as a phyto-remediating agent and it also allows the production of large amounts of cellulose and hemicellulose (Fiorentino et al. 2010, 2013).

Many studies have shown that sugar polymers (cellulose and hemicellulose) present in biomasses can be hydrolyzed into fermentable sugars and then converted into fuels and chemicals (Wettstein et al. 2012; Kobayashi and Fukuoka 2013; Kajaste 2014). The Pacific Northwest National Laboratory (PNNL) and National Renewable Energy Laboratory (NREL) identified 12 building blocks produced through sugar conversions which could be used to obtain a variety of high-value biobased products. These include succinic acid, glucaric acid, aspartic acid, glycerol, sorbitol and xylitol/arabinitol (Hermann et al. 2007).

The production of bioproducts from lignocellulosic biomass requires several steps (Kumar et al. 2009), including an initial pretreatment step needed to break the lignin barrier and make cellulose and hemicellulose accessible to the enzymes during the hydrolysis to produce fermentable sugars. Enzymatic hydrolysis of sugar polymers using biomass-degrading enzymes such as cellulase and hemicellulase is preferred over dilute acid hydrolysis because of the higher conversion and lower environmental impact (Taherzadeh and Keikhosro 2007). Due to the complexity of the polysaccharides of the pretreated biomass, a tailor-made enzyme cocktail is required to achieve a more efficient saccharification (Gao et al. 2010a, 2011). Besides the enzymes involved in the hydrolysis of cellulose and the backbone of hemicellulose, some other enzymes with hydrolytic ability towards glycoside branches in hemicellulose were proven to improve the overall monosaccharide yield.

This study was aimed at evaluating the conversion of *A. donax* into monosaccharides for bioethanol or bioproducts production by enzymatic hydrolysis of the polysaccharides issued from pretreatment of the biomass by ammonia fibre expansion (AFEX). To the best of our knowledge, this is the first manuscript on saccharification of AFEX-pretreated *A. donax*.

In particular, the capacity of the cellulase CelStrep from *Streptomyces* sp. G12 recombinantly expressed in *Escherichia coli* (Amore et al. 2012b) and α-l-arabinofuranosidase PoAbf from the fungus *Pleurotus ostreatus* recombinantly expressed in *Pichia pastoris* (Amore et al. 2012a) and its evolved mutant rPoAbf F435Y/Y446F (Giacobbe et al. 2014) to improve the sugar yields of AFEX-pretreated *A. donax* was investigated. The rPoAbf F435Y/Y446F mutant had been previously developed with the aim to improve the catalytic efficiency of the investigated α-l-arabinofuranosidase (Giacobbe et al. 2014). It was further investigated in biomass conversion due to its ability to hydrolyze both soluble and insoluble substrates better than that of the rPoAbf wild type. Differently from our previous work (Marcolongo et al. 2014), the investigated enzymes were used in combination with purified fungal and bacterial enzymes to define a tailor-made enzyme cocktail for *A. donax* hydrolysis in order to reduce the cost of the enzymatic saccharification process.

## Materials and methods

### Feedstock

Biomasses used in this study for pretreatment and saccharification experiments include corn stover and the giant reed (*A. donax* L.). *A. donax* was produced in marginal lands of southern Italy with a low-input cropping system (Forte et al. 2015), and corn stover was provided by Michigan State University (MSU). The biomasses were milled with a 2-mm-diameter sieve and stored under dry conditions at room temperature until further use. The moisture content was measured using a moisture analyser (Sartorius MA35M, Elk Grove, IL).

### Compositional analysis

Compositional analyses of *A. donax* and corn stover biomasses were performed following the National Renewable Energy Laboratory (NREL) Laboratory Analytical Procedures (LAPs) standard protocols:

- “Preparation of samples for compositional analysis” (Hames et al. 2008)
- “Determination of structural carbohydrates and lignin in Biomass”
- “Determination of Total Solids in Biomass and Total Dissolved Solids in Liquid Process Samples” (Sluiter et al. 2008)
- “Determination of Ash in Biomass” (Sluiter et al. 2005b)
- “Determination of structural carbohydrates and lignin in biomass” (Sluiter et al. 2005a)

Monomeric sugars were quantified using a Bio-Rad Aminex HPX-87H high-performance liquid chromatography (HPLC) column using 5 mM sulphuric acid as mobile phase.

The variability between experiments is reflected by the standard deviation reported.

### AFEX pretreatment

Corn stover and *A. donax* were pretreated through the AFEX method by varying the ammonia to biomass ratio (1:1 and 2:1), reaction temperature (100–160 °C), moisture (60–233 % on dry weight basis) and fixed residence time (15 min). AFEX was carried out in a high-pressure stainless steel vessel. Biomass was first loaded into the vessel with appropriate moisture after taking into consideration the moisture content of original biomass. Then, the reactor was closed and vacuum applied to remove residual air in the reactor. The required amount of liquid ammonia was loaded into the reactor using an ammonia delivery pump. The vessel was heated by an external mantle and the biomass was mixed during the AFEX process for 15 min. As the temperature of the reactor increased, the pressure in the vessel rose (between 200 and 400 psi) depending on the ammonia to biomass loading. The pressure was released from the vessel and ammonia was vented in a fume hood. The pretreated biomass was moved to a tray and dried in a fume hood overnight to remove residual ammonia. Then, dry AFEX-treated biomass was stored in a sealed polythene bag at 4 °C until further use.

### Purified enzymes and their source

The following purified enzymes were tested in this study:

- Fungal cellobiohydrolase I (CBH I; glycoside hydrolase (GH) family 7A), cellobiohydrolase II (CBH II; GH family 6A) and endoglucanase I (EG I; GH family 7B) were purified from Spezyme CP using several chromatography steps (size-exclusion chromatography, anion and cation exchange chromatography, hydrophobic interaction chromatography and affinity chromatography) as described in Gao et al. (2010a). The enzymes were used at a concentration of 3.32 mg/g glucan each.
- Fungal β-glucosidase (βG; GH family 3) was purified from Novozyme 188 using anion exchange chromatography followed by cation exchange chromatography as described in Gao et al. (2010a) and used at a concentration of 2 mg/g glucan.
- Bacterial xylanases (LX3, GH family 10; LX4, GH family 11) from *Clostridium thermocellum* library (Gao et al. 2011) (bacterial cell concentrate was a kind gift of Dr. Paul Weimer, United States Department of Agriculture, Agricultural Research Service, United States Dairy Forage Research Center, WI, USA) were recombinantly expressed in *E. coli* and purified using HIS-select nickel affinity chromatography. The enzymes were used at a concentration of 1.66 mg/g glucan each.
- Bacterial β-xylosidase (LβX; GH family 52) from *Geobacillus stearothermophilus* XYNB2 (Gao et al. 2010b) was recombinantly expressed in *E. coli* and purified using HIS-select nickel affinity chromatography. The enzyme was used at 0.6 mg/g glucan.
- Bacterial α-arabinofuranosidase (LarbF, GH family 51) from *Geobacillus* sp. G11MC16 was recombinantly expressed in *E. coli* and purified using HIS-select nickel affinity chromatography. The enzyme was loaded at 0.6 mg/g glucan (Gao et al. 2011).
- Bacterial CelStrep (EMBL accession number HE862416) from *Streptomyces* sp. G12 was recombinantly expressed in *E. coli* (Amore et al. 2012b). The enzyme was purified by ammonium sulphate precipitation followed by hydrophobic interaction chromatography, as described in Amore et al. (2012b) and used at 3.32 mg/g glucan.
- Fungal α-l-arabinofuranosidase (rPoAbf) from *P. ostreatus* (ATCC 66376) was recombinantly expressed in *P. pastoris* (Amore et al. 2012a). The enzyme was purified by ammonium sulphate precipitation followed by hydrophobic interaction chromatography, as described in Amore et al. (2012a) and loaded at 0.6 mg/g glucan.
- The variant F435Y/Y446F of PoAbf, previously selected from a directed evolution library of rPoAbf, was recombinantly expressed in *P. pastoris* (Giacobbe et al. 2014), purified as rPoAbf wild type and loaded at 0.6 mg/g glucan.

### Determination of protein concentration

Purified protein concentration was determined by the Pierce (Pierce Biotechnology, Rockford, USA) bicinchoninic acid (BCA) assay kit following the manufacturer’s instructions. Bovine serum albumin (BSA) was used as standard.

### Enzymatic hydrolysis

The bioconversion experiments were performed in five vials at 1 % (*w*/*w*) glucan loading in 50 mM citrate buffer (pH 4.8) with the desired enzymes. 0.5 mM sodium azide was used to prevent microbial and fungal growth. The saccharification was performed at 50 °C and 250 rpm in a shaking incubator. Sampling was done every 24 h to evaluate the sugar composition using high-performance liquid chromatography (HPLC) system. The commercial enzyme mixture Novozymes Cellic® (60 % Ctec3 and 40 % Htec3) was used (at a loading of 15 mg/g of glucan) for saccharification experiments to evaluate the best pretreatment conditions. The hydrolysis of AFEX-treated biomasses was performed with the following enzymes at a loading of around 16.5 mg/g glucan (Table 1). An enzymatic mixture named mix A was prepared, including CBH I, cellobiohydrolase II, EG I at a concentration of 3.32 mg/g glucan each, βG 2 mg/g glucan, xylanases (LX3, LX4) 1.66 mg/g glucan each and LβX and LarbF 0.6 mg/g glucan each. In mix B, the EG I was replaced by cellulase rCelStrep; in mix C, the LarbF was replaced by the rPoAbf; in mix D, the LarbF was replaced by a mutant of rPoAbf named rPoAbf F435Y/Y446F; in mix E, both EGI and LarbF were replaced by rCelStrep and rPoAbf; in mix F, both EGI and LarbF were replaced by rCelStrep and rPoAbf F435Y/Y446F. Moreover, rCelStrep (3.32 mg/g glucan), rPoAbf (0.6 mg/g glucan) and rPoAbf mutant (0.6 mg/g glucan) were added to the commercial mixture Novozymes Cellic®.

**Table 1:** Mixtures of bacterial and fungal enzymes tested on AFEX-treated biomasses

Individual enzyme loading (mg/g glucan)											
Mix	CBHI	CBHII	βG-	LX3	LX4	LβX	EGI	LarbF	rCelstrep	rPoAbf	rPoAbf F435Y/Y446F
A	3.32	3.32	2	1.66	1.66	0.6	3.32	0.6			
B	3.32	3.32	2	1.66	1.66	0.6		0.6	3.32		
C	3.32	3.32	2	1.66	1.66	0.6	3.32			0.6	
D	3.32	3.32	2	1.66	1.66	0.6	3.32				0.6
E	3.32	3.32	2	1.66	1.66	0.6		0.6	3.32	0.6	
F	3.32	3.32	2	1.66	1.66	0.6		0.6	3.32		0.6

### Sugar analysis

About 200 μl hydrolysate collected at different times during enzyme hydrolysis was transferred to a centrifuge tube, heated to 100 °C for 10 min (to denature the enzymes), centrifuged at 8000 rpm for 10 min to remove the precipitates and then stored in a HPLC vial at −20 °C until further use. A Bio-Rad Aminex HPX-87P HPLC column was used to determine the monomeric sugar concentrations in the hydrolysate. All experiments were performed in triplicate. Shimadzu HPLC Prominence system (Columbia, MD, USA) with a refractive index detector (RID), was employed for analysing the sugars. Water was the mobile phase at a fixed flow rate of 0.6 ml/min, with isocratic elution. The column temperature was maintained at 60 °C, and the HPLC sample injection volume was 20 μl. Standard curves were generated using different concentrations of mixed sugars. A guard column with similar packing was used throughout the chromatography experiments.

### Statistical analyses

One-way ANOVA followed by Tukey’s HSD post hoc for pairwise comparison of means (at *P* ≤ 0.05) was used to assess the difference in the sugar conversion of the different enzyme mixtures used to hydrolyze *A. donax* or corn stover biomass. Statistical analyses were performed using SPSS 13.0 statistical software package (SPSS Inc., Cary, NC, USA).

## Results

### Characterization of biomasses

Analyses of the macromolecular composition of the un-pretreated biomasses *A. donax* and corn stover were performed, and the results, reported in Table 2, revealed that *A. donax* contains 61.41 % of structural polysaccharides, 26.24 % Klason lignin and 4.9 % ash, while corn stover contains 63.23 % of structural polysaccharides, 20.06 % Klason lignin and 6.17 % ash. Both the analysed biomasses contain significant percentages of C5 sugars mainly represented by xylan (∼20 %) while arabinan represents only around 2 % of total dry weight and the C6 sugars consist mainly of glucan (>37 %).

**Table 2:** *A. donax* and corn stover composition before AFEX pretreatment (standard deviation obtained from three independent experiments)

Composition	*A. donax* (%, dry weight basis)	Corn stover (%, dry weight basis)
Moisture content	6.0 ± 0.5	8.7 ± 0.6
Ash	4.9 ± 0.6	6.7 ± 1.0
Structural carbohydrate		
Glucan	38.0 ± 0.0	38.41 ± 0.1
Xylan	21.0 ± 0.1	19.9 ± 1.0
Galactan	1 ± 0.1	1.4 ± 0.1
Arabinan	1.5 ± 0.0	2.5 ± 0.2
Mannan	N.D.	N.D.
Lignin		
Acid-insoluble lignin	26.2 ± 0.1	20.1 ± 0.7
Acid-soluble lignin	1.9 ± 0.0	2.0 ± 0.3

*ND* Not determined

### Selection of AFEX pretreatment conditions

The yield of monosaccharides released during hydrolysis depends on various AFEX parameters such as moisture content, ammonia to biomass loading, residence time and temperature. Therefore, in order to assess the best pretreatment conditions for *A. donax*, three different AFEX treatments were carried out (Table 3). The AFEX-tested conditions, condition 1, condition 2 and condition 3 reported in Table 3, were chosen since they had been previously identified as the best conditions for the AFEX pretreatment of *Miscanthus* x *giganteus* (Murnen et al. 2007), corn stover (Balan et al. 2009b) and switchgrass (Bals et al. 2010), respectively. Since corn stover was used as the reference biomass in this study, the best AFEX condition for pretreatment of this biomass was tested to pretreat *A. donax*. Nevertheless, since *M.* x *giganteus* and switchgrass are perennial crops like *A. donax*, the best conditions for their AFEX pretreatment were also tested. The samples of *A. donax* pretreated in the different conditions were then enzymatically hydrolyzed for 168 h using a commercial enzymatic preparation consisting of Novozymes Cellic®, 60 % Ctec3 and 40 % Htec3. In Table 3, the glucan and xylan conversions obtained after 24, 72 and 168 h of enzymatic hydrolysis of *A. donax* after the three different AFEX pretreatments are reported in comparison to corn stover. Both glucan and xylan hydrolyses reached their maximum level with AFEX pretreatment condition 2: 130 °C, 1 kg ammonia/1 kg dry biomass and 60 % of moisture content. Based on these data, this condition was selected for further hydrolysis experiments.

**Table 3:** Experimental AFEX pretreatment conditions tested in this study and percentage of glucan and xylan conversion after 24, 72 and 168 h hydrolysis of *A. donax* and corn stover by the commercial enzymatic preparation consisting of Novozymes Cellic® 60 % Ctec3 and 40 % Htec3 (15 mg/g glucan)

*Arundo donax*										
AFEX conditions	Ammonia loading (kg/kg dry biomass)	Temperature (°C)	Moisture content (%)	Time (min)	Glucan conversion (%)			Xylan conversion (%)		
					24 h hydrolysis	72 h hydrolysis	168 h hydrolysis	24 h hydrolysis	72 h hydrolysis	168 h hydrolysis
1	1:1	100	80	15	34.4	41.2	49.7	55.6	60.0	66.9
2	1:1	130	60	15	39.0	44.1	56.8	66.4	65.9	75.5
3	2:1	160	233 (water soak)	15	22.4	24.0	28.3	22.4	24.5	29.6
Corn stover										
	Ammonia loading (kg/kg dry biomass)	Temperature (°C)	Moisture content (%)	Time (min)	Glucan conversion (%)			Xylan conversion (%)		
					24 h hydrolysis	72 h hydrolysis	168 h hydrolysis	24 h hydrolysis	72 h hydrolysis	168 h hydrolysis
	1:1	130	60	15	71.9	79.1	90.4	70.0	90.0	100.0

### Saccharification of *A. donax* and corn stover using purified enzyme cocktail and commercial enzyme cocktail

**Fig. 1:**
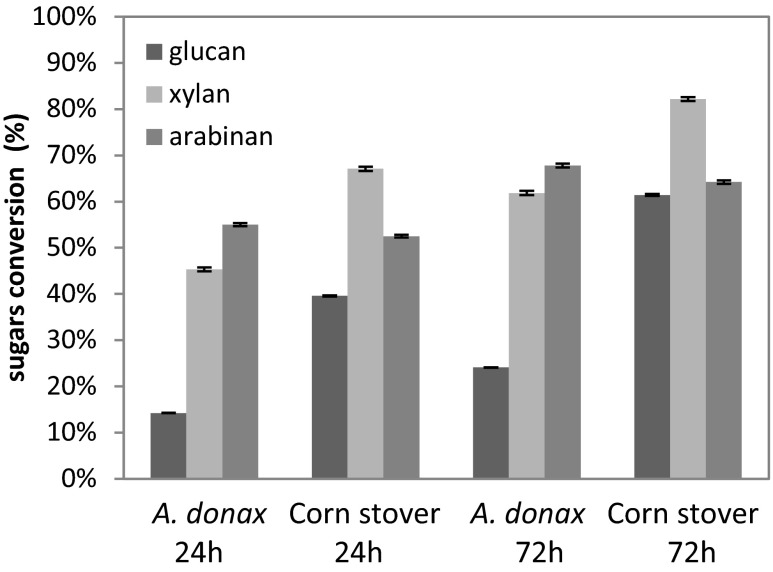
Glucan, xylan and arabinan conversion after 24 and 72 h of hydrolysis on AFEX-treated corn stover and *A. donax* using the enzyme mix A containing the cellulases CBH I, CBH II and EG I loaded at 3.32 mg/g glucan each, βG loaded at 2 mg/g glucan, and the endoxylanases LX3 and LX4 and accessory hemicellulases LarbF and LβX loaded at 1.66 and 0.6 mg/g glucan each, respectively

The enzymatic hydrolysis of biomass using commercial enzymes is not appropriate to evaluate the role of individual enzymes involved in the process, and it is difficult to obtain a tailor-made enzyme cocktail for the biomass of interest. Previous works have shown that an optimal cocktail of cellulolytic enzymes (CBH I, CBH II, EGI, βG) and a set of hemicellulases and accessory enzymes (LX3, LX4, LarbF, LβX ) are required to improve the hydrolysis of AFEX-treated corn stover (Gao et al. 2010a, 2011). Based on these results, the enzyme mix A (CBH I, CBH II, EGI, βG, LX3, LX4, LarbF, LβX), previously optimized for corn stover (Gao et al. 2011), was used as reference enzymatic mixture to hydrolyze *A. donax*. Figure 1 shows the glucan, xylan and arabinan conversion percentages from corn stover and *A. donax* after AFEX treatment in selected condition 2, by 24- and 72-h-long hydrolyses with mix A.

As far as *A. donax* is concerned, although the enzyme mix A gave similar xylan hydrolysis (∼63 % after 72 h) compared with the commercial enzymatic preparation, Novozymes Cellic®, 60 % Ctec3 and 40 % Htec3, reported in Table 2, lower glucan hydrolysis was achieved (less than 30 % after 72 h). As regards corn stover, mix A gave sugar conversions (Fig. 1) similar to those obtained with the Novozyme Cellic® enzyme cocktail (Table 3).

### Effect of the enzymes rCelStrep, rPoAbf and its variant on saccharification of *A. donax* and corn stover in comparison with the enzymes EGI or LarbF

The effect of the arabinofuranosidase from the fungus *P. ostreatus* recombinantly expressed in *P. pastoris* rPoAbf, its evolved mutant expressed in the same yeast rPoAbf F435Y/Y446F and the cellulase from *Streptomyces* sp. *G12* rCelStrep recombinantly expressed in *E. coli* in bioconversion of AFEX-pretreated *A. donax* and corn stover was tested. In these experiments, the enzyme mix A, containing cellulases (CBHI, CBHII, EGI and βG), endoxylanases (LX3, LX4) and accessory hemicellulases (LarbF and LβX), was used as reference enzyme mixture, and the effect of substituting EGI with rCelStrep and LarbF with rPoAbf or rPoAbf F435Y/Y446F was assessed. In more detail, the bacterial cellulase rCelStrep was used instead of the fungal endoglucanase EGI in the enzyme mix B while the fungal arabinofuranosidases rPoAbf was used instead of the bacterial arabinofuranosidase LarbF in the enzyme mix C. The evolved variant of rPoAbf (rPoAbf F435Y/Y446F) was used instead of the bacterial arabinofuranosidase LarbF in the enzyme mix D due to its ability to hydrolyze both soluble and insoluble substrates better than that of the rPoAbf wild type. Moreover, rCelStrep was also tested in combination with rPoAbf (mix E) or its variant (mix F) to check a possible synergism between bacterial and fungal enzymes.

Table 4 summarizes the conversion results of AFEX-pretreated *A. donax* biomass using different purified enzymatic mixtures. The sugar conversion results showed that the substitution of EGI with rCelstrep (mix B) gave significantly higher glucan conversion (28 %) after 72 h than that obtained with enzyme mix A (24.1 %), corresponding to 15 % increase in sugar conversion. On the other hand, the use of rCelstrep (mix B) decreased arabinan hydrolysis, giving an arabinan conversion 17 % lower than that obtained with the enzyme mix A after 72 h.

**Table 4:** *A. donax* sugar conversion in 50 mM citrate buffer pH 4.5 and *T* 50 °C, for 24 and 72 h. The values represent the means ± SD of three replicates. Different superscript letters after the values indicate significant differences (*P* ≤ 0.05) on the same column within samples collected after 24 or 72 h

Enzyme mixture	Purified enzyme cocktail	Glucan conversion (%)	Xylan conversion (%)	Arabinan conversion (%)
24 h hydrolysis				
Mix A	CBHI-CBHII-βG-LX3-LX4-LβX + EGI & LarbF	14.2 ± 1.6^ab^	45.4 ± 0.5^a–c^	55.0 ± 2.5^b^
Mix B	CBHI-CBHII-βG-LX3-LX4-LβX + rCelstrep & LarbF	11.8 ± 2.9^a^	43.7 ± 2.5^ab^	52.3 ± 2.1^b^
Mix C	CBHI-CBHII-βG-LX3-LX4-LβX + EGI & rPoAbf	16.9 ± 0.2^bc^	49.2 ± 0.5^cd^	56.1 ± 4.0^b^
Mix D	CBHI-CBHII-βG-LX3-LX4-LβX + EGI & rPoAbf F435Y/Y446F	17.5 ± 1.8^c^	52.5 ± 4.2^d^	64.9 ± 6.1^c^
Mix E	CBHI-CBHII-βG-LX3-LX4-LβX + rCelstrep & rPoAbf	16.6 ± 0.3^bc^	41.8 ± 0.1^a^	19.9 ± 0.3^a^
Mix F	CBHI-CBHII-βG-LX3-LX4-LβX + rCelstrep & rPoAbf F435Y/Y446F	16.4 ± 0.1^bc^	46.1 ± 0.8^bc^	53.1 ± 1.4^b^
72 h hydrolysis				
Mix A	CBHI-CBHII-βG-LX3-LX4-LβX + EGI & LarbF	24.1 ± 1.1^b^	61.8 ± 0.9^b^	67.8 ± 0.6^c^
Mix B	CBHI-CBHII-βG-LX3-LX4-LβX + rCelstrep & LarbF	28.5 ± 2.6^c^	59.1 ± 6.2^b^	56.3 ± 0.3^b^
Mix C	CBHI-CBHII-βG-LX3-LX4-LβX + EGI & rPoAbf	24.6 ± 1.5^b^	61.5 ± 0.9^b^	57.8 ± 2.6^b^
Mix D	CBHI-CBHII-βG-LX3-LX4-LβX + EGI & rPoAbf F435Y/Y446F	61.6 ± 0.4^d^	62.7 ± 4.1^b^	78.2 ± 8.2^d^
Mix E	CBHI-CBHII-βG-LX3-LX4-LβX + rCelstrep & rPoAbf	20.6 ± 1.2^a^	48.5 ± 0.7^a^	27.9 ± 0.7^a^
Mix F	CBHI-CBHII-βG-LX3-LX4-LβX + rCelstrep & rPoAbf F435Y/Y446F	22.1 ± 1.2^ab^	57.5 ± 1.4^b^	60.9 ± 4.0^bc^

When the rPoAbf wild type was used instead of LarbF (mix C), no significant differences in glucan and xylan hydrolyses were observed. The use of rPoAbf wild type gave an arabinan hydrolysis 14 % significantly lower than that obtained with mix A after 72 h hydrolysis.

**Fig. 2:**
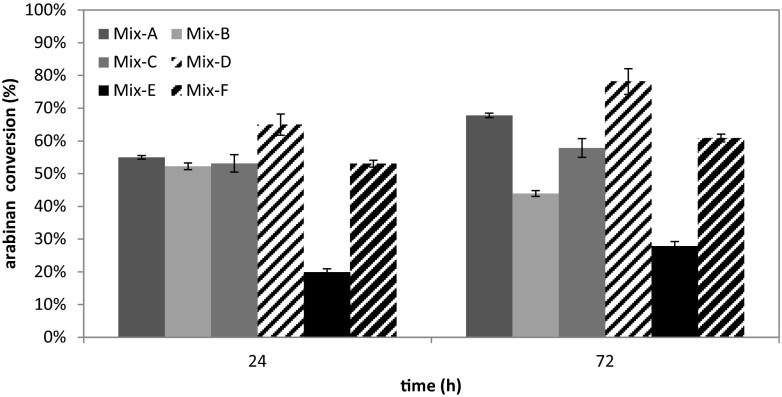
Arabinan conversion after 24 and 72 h hydrolysis of ammonia fibre expansion (AFEX)-treated *A. donax* with the enzymatic mixtures reported in Table 4

The use of rPoAbf F435Y/Y446F instead of LarbF (enzyme mix D in comparison with enzyme mix A) and the wild-type rPoAbf (mix D in comparison with mix C) led to improvements in sugar conversion. The use of mix D gave 60 % glucan conversion after 72 h, which is 2.5 times higher than that reached with mix A and mix C. The use of mix D gave 53 % xylan conversion after 24 h, which is 16 % higher than that obtained with mix A. Moreover, the xylan conversion after 72 h saccharification with mix D achieved 63 %, similar to that obtained with mix A and mix C. The arabinan conversion was found to be 78 % after 72 h, which is 15 and 26 % higher than that obtained with the mix A and mix C, respectively (Fig. 2).

The effect of rCelstrep in combination with rPoAbf (mix E) and rPoAbf F435Y/Y446F (mix F) was also analysed (Table 4). After 24 h, glucan conversion was found to be not significantly higher than mix A, for both mix E and mix F. However, about 20 % glucan conversion was obtained after 72 h for enzyme mix E, which is 14.5 % lower than that obtained with enzyme mix A; also, the xylan conversion resulted significantly lower than that with enzyme mix A after 72 h hydrolysis. As regards mix F, no significant differences with enzyme mix A were found in xylan hydrolysis after both 24 and 72 h. Mix E gave 20 and 28 % arabinan conversion, which are 64 and 56 % lower than that with enzyme mix A, after 24 and 72 h, respectively. On the other hand, no significant differences in arabinan conversion were obtained with mix F in comparison to mix A.

Hydrolysis of AFEX-treated corn stover using purified enzyme cocktail mixtures showed that no significant increase in sugar conversion was achieved replacing enzyme mix A with mix B, mix E and mix F (Table 5). On the other hand, enzyme mix C and mix D gave 8 % higher glucan conversion after 24 h when compared to enzyme mix A. However, the sugar conversion decreased after 72 h hydrolysis. Enzyme mix C and mix D gave 57.4 and 63.2 % arabinan conversion, respectively, after 24 h, which is 8.5 and 17 % higher than that with mix A.

**Table 5:** Corn stover sugar conversion in 50 mM citrate buffer pH 4.5, *T* 50 °C. The values represent the means ± SD of three replicates. Different superscript letters after the values indicate significant differences (*P* ≤ 0.05) on the same column within samples collected after 24 or 72 h

Enzyme mixture	Purified enzyme cocktail	Glucan conversion (%)	Xylan conversion (%)	Arabinan conversion (%)
24 h hydrolysis				
Mix A	CBHI-CBHII-βG-LX3-LX4-LβX + EGI & LarbF	39.6 ± 0.8^c^	67.1 ± 1.2^c^	52.5 ± 1.3^b^
Mix B	CBHI-CBHII-βG-LX3-LX4-LβX + rCelstrep & LarbF	32.7 ± 0.1^a^	67.5 ± 0.1^c^	53.5 ± 0.1^bc^
Mix C	CBHI-CBHII-βG-LX3-LX4-LβX + EGI & rPoAbf	42.8 ± 0.7^d^	72.8 ± 1.1^d^	57.4 ± 0.8^d^
Mix D	CBHI-CBHII-βG-LX3-LX4-LβX + EGI & rPoAbf F435Y/Y446F	42.7 ± 0.2^d^	73.6 ± 0.4^d^	63.2 ± 0.2^e^
Mix E	CBHI-CBHII-βG-LX3-LX4-LβX + rCelstrep & rPoAbf	38.3 ± 1.1^b^	59.6 ± 1.2^a^	54.1 ± 2.0^c^
Mix F	CBHI-CBHII-βG-LX3-LX4-LβX + rCelstrep & rPoAbf F435Y/Y446F	39.2 ± 0.7^c^	65.5 ± 1.1^b^	16.4 ± 1.2^a^
72 h hydrolysis				
Mix A	CBHI-CBHII-βG-LX3-LX4-LβX + EGI & LarbF	61.4 ± 1.2^f^	82.2 ± 1.5^e^	64.2 ± 2.0^e^
Mix B	CBHI-CBHII-βG -LX3-LX4-LβX + rCelstrep & LarbF	56.3 ± 0.7^e^	83.6 ± 2.1^e^	14.4 ± 0.2^a^
Mix C	CBHI-CBHII-βG -LX3-LX4-LβX + EGI & rPoAbf	11.7 ± 1.0^a^	77.0 ± 1.2^d^	56.0 ± 0.1^c^
Mix D	CBHI-CBHII-βG -LX3-LX4-LβX + EGI & rPoAbf F435Y/Y446F	41.2 ± 0.6^b^	73.4 ± 0.4^c^	63.5 ± 0.2^e^
Mix E	CBHI-CBHII-βG -LX3-LX4-LβX + rCelstrep & rPoAbf	47.2 ± 0.8^c^	57.9 ± 0.2^a^	59.2 ± 1.4^d^
Mix F	CBHI-CBHII-βG -LX3-LX4-LβX + rCelstrep & rPoAbf F435Y/Y446F	48.7 ± 1.2^d^	65.5 ± 0.5^b^	27.2 ± 0.2^b^

It is worth noting that the evolved variant rPoAbf F435Y7Y446F gave a better arabinan conversion than the wild-type enzyme for both the biomasses.

### Synergy between commercial enzyme cocktail and purified enzymes (rPoAbf and/or rCelstrep)

To study synergy between the commercial enzyme cocktail Novozymes Cellic®, Ctec3 and Htec3, and the purified enzymes rPoAbf, its evolved mutant rPoAbf F435Y/Y446F and rCelstrep, hydrolysis of AFEX-pretreated *A. donax* and corn stover was carried out adding each tested enzyme or their combinations (rCelstrep and rPoAbf or rCelstrep and rPoAbf F435Y/Y446F) to the Novozymes cocktail.

**Fig. 3:**
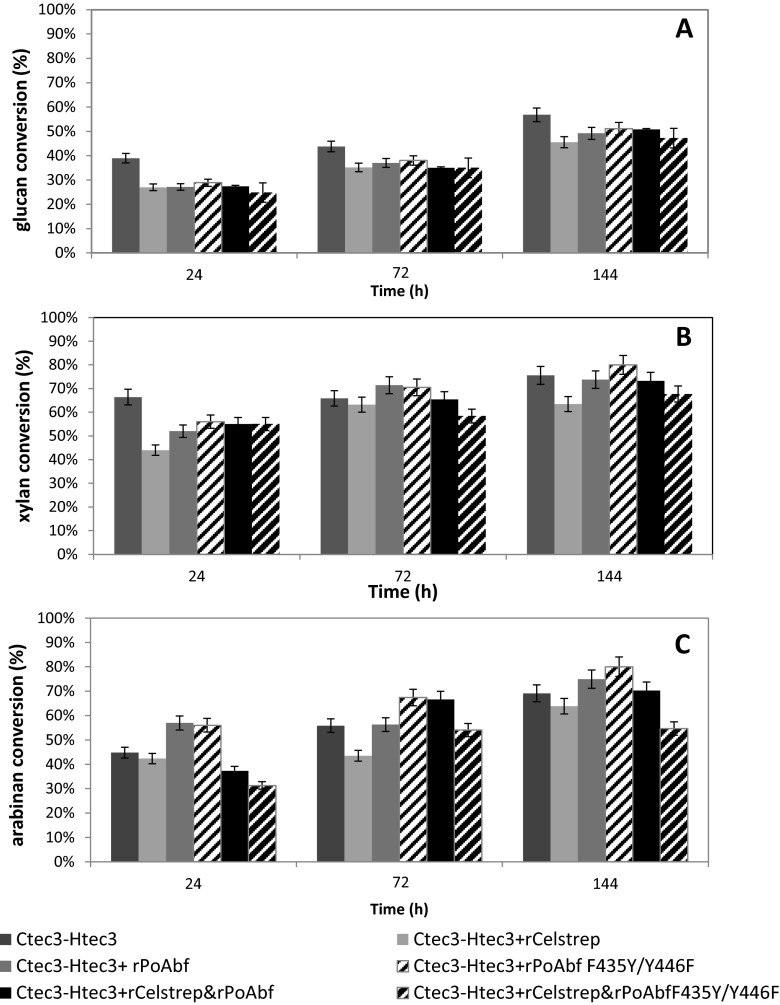
Glucan (**a**), xylan (**b**) and arabinan (**c**) conversion of AFEX-treated *A. donax* achieved using commercial enzyme preparation Novozymes Cellic®, 60 % Ctec3 and 40 % Htec3, with or without addition of rPoabf, its variant F435Y/Y446F, rCelstrep or a combination of these enzymes

The glucan conversion using commercial enzyme cocktail was found to be higher when compared to supplementation with wild-type rPoAbf or its evolved variant or rCelstrep or both enzymes (Fig. 3a). These results could be explained by hypothesizing an anti-synergism between the activity of (hemi)cellulases in commercial mixture and purified ones. The negative level of synergism was already described for commercial enzymes involved in biomass degradation and purified ones (Woodward J 1991; Morrison 2014).

Supplementing rPoAbf and its evolved variant gave 70 % xylan conversion, 7 % higher than that obtained by the commercial preparation. After 6 days of hydrolysis, 80 % xylan conversion was achieved when rPoAbf F435Y/Y446F was used along with commercial enzymes (Fig. 3b). The arabinan conversion was also improved by supplementing rPoAbf or variant to the commercial enzyme cocktail by 27 and 36 %, respectively, after 24 h of hydrolysis (Fig. 3c). A maximum arabinan conversion of 80 % was achieved after 6 days of hydrolysis when rPoAbf was added to commercial enzymes.

**Fig. 4:**
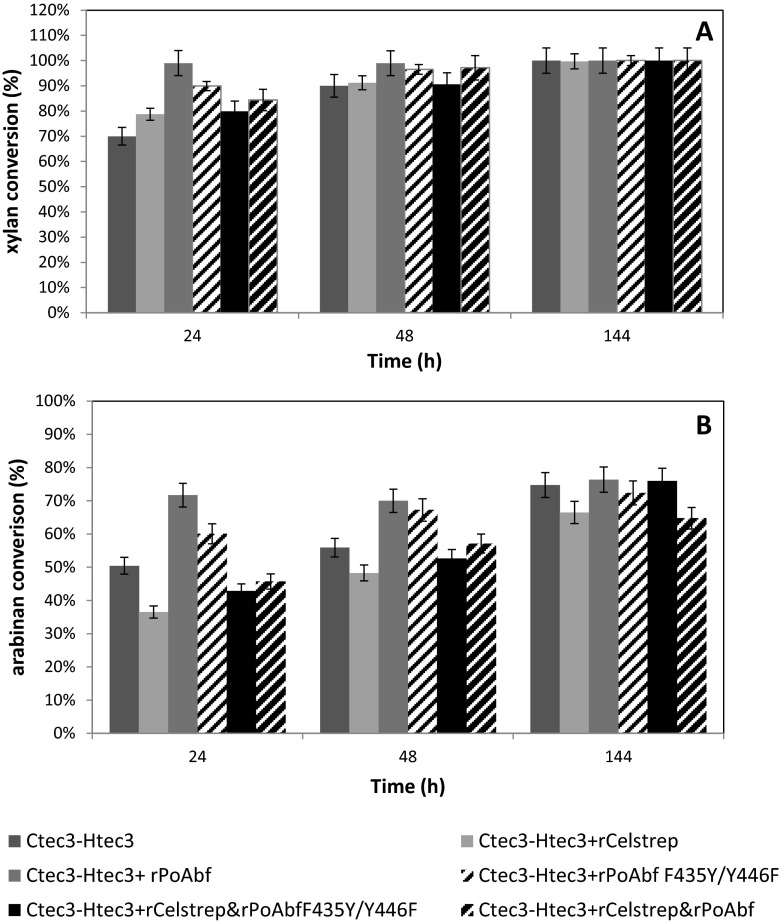
Xylan (**a**) and arabinan (**b**) conversion of AFEX-treated corn stover achieved using commercial enzyme preparation Novozymes Cellic®, 60 % Ctec3 and 40 % Htec3, with or without addition of rPoabf, its variant F435Y/Y446F, rCelstrep or a combination of these

In the case of corn stover, no appreciable increase in glucan conversion was observed when commercial preparation was supplemented with wild-type rPoAbf or its variant or rCelstrep or both enzymes (data not shown). However, 99 % xylan conversion was achieved after 24 h of hydrolysis with commercial enzymes supplemented with rPoAbf wild type (Fig. 4a). The same xylan conversion was achieved using commercial preparation and commercial preparation in combination with rPoAbf F435Y/Y446F or rCelstrep or both enzymes after 6 days of hydrolysis. Supplementation of commercial enzyme cocktail with rPoAbf gave 70 % of arabinan conversion after 24 h of hydrolysis (Fig. 4b).

## Discussion

This study was focused on pretreatment and saccharification of the giant reed (*A. donax* L.) using corn stover as reference lignocellulosic biomass. *A. donax* is a biomass crop that can be cultivated with high yields in marginal areas not suitable for the traditional food crops, gaining at the same time several environmental benefits such as soil protection from erosion, C storage into the soil and phytoremediation of polluted soils (Fagnano et al. 2015; Forte et al. 2015).

The data of macromolecular composition of *A. donax* and corn stover used in this study was revealed to be in agreement with data reported in previous studies (Caparrós et al. 2007; Li et al. 2011; Scordia et al. 2012).

The pretreatment methods so far tested and reported in literature for *A. donax* include the steam explosion, acid-catalyzed steam explosion and ammonia soaking (De Bari et al. 2013; Van Den Brink et al. 2013; Marcolongo et al. 2014). However, most of these methods solubilize large portions of xylan from this biomass.

On the other hand, ammonia fibre expansion (AFEX) is a dry to dry process which preserves all the original carbohydrates (Gao et al. 2011 and 2013; Li et al. 2011; Harun et al. 2013; Uppugundla et al. 2014) without any loss. This pretreatment has never been used for *A. donax*. Therefore, this pretreatment process was selected for this study, in order to avoid the loss of the significant amounts of xylan present in the investigated biomass (Table 2).

Corn stover was chosen as reference lignocellulosic biomass because AFEX pretreatment had been previously carried out on it in several works giving high yields of both glucose and xylose (Balan et al. 2009; Li et al. 2011). It is worthy of note that the best AFEX condition selected in this study for the biomass of *A. donax* (condition 2: 130 °C, 1 kg ammonia/1 kg dry biomass and 60 % of moisture content) was the same pretreatment condition previously adopted for corn stover (Gao et al. 2011).

The effect of the α-l-arabinofuranosidase rPoAbf, its evolved mutant rPoAbf F435Y/Y446F and the cellulase rCelStrep, to enhance the saccharification of the pretreated giant reed (*A. donax* L.) in substitution of the purified enzymes LarbF or EGI, respectively, was tested in bioconversion of AFEX-pretreated *A. donax*. Moreover, synergy between commercial enzyme cocktail Novozymes Cellic®, Ctec3 and Htec3, and rPoAbf, rPoAbf F435Y/Y446F and rCelStrep in hydrolysis of AFEX-pretreated *A. donax* and corn stover was also investigated. Corn stover was the reference feedstock for all the saccharification experiments.

As the most positive effects observed for *A. donax* conversion, the use of the rPoAbf F435Y/Y446F within the mixture of purified enzymes allowed achieving better glucan, xylan and arabinan conversions than LarbF and rPoAbf wild type. In particular, rPoAbf F435Y/Y446F gave a glucan conversion after 72 h 2.5 times higher than that obtained with the other enzymes, a xylan conversion after 24 h 16 % higher than that obtained with LarbF and an arabinan conversion after 72 h 15 and 26 % higher than that obtained with LarbF and the wild-type rPoAbf, respectively. The improvement of glucan conversion with the enzymatic cocktail containing rPoAbf F435Y/Y446F can be explained with the enhancement of the accessibility of this polysaccharide due to xylan hydrolysis, as previously reported (Polizeli et al. 2005).

The higher glucan conversion yield obtained substituting EGI with rCelstrep in the mixture of purified enzymes suggests a synergistic role of the bacterial cellulase rCelstrep when mixed with the fungal cellulases CBHI, CBHII and βG on glucan conversion. The use of Celstrep in combination with rPoAbf and its variant gave lower sugar conversion than those achieved using rPoAbf and its variant in combination with EGI, which could indicate a lower synergism between bacterial cellulase and fungal arabinofuranosidase than that between fungal cellulase and arabinofuranosidase. Moreover, this effect was more pronounced at longer times, which could be due to the loss of degree of synergy during the hydrolysis caused by structural modifications of the substrate as reported by Andersen et al. (2008) or saturation of sites for enzyme activity as proposed by Boisset et al. (2001).

As regards corn stover biomass, no significant improvement of sugar conversion was observed when the reference mixture of purified enzymes (mix A previously optimized for this biomass by Gao et al. 2011) was replaced by the other purified enzymes tested in this work.

The comparison of the effects of rPoAbf wild type and rPoAbf F435Y/Y446F in the presence of rCelstrep showed that the use of rPoAbf wild type led to the highest arabinan conversion from corn stover (mix E), while for *A. donax* biomass, the best conversion was obtained using rPoAbf F435Y/Y446F (mix F). These results could be explained by hypothesizing a diverse specificity and synergism of the enzymes towards the different biomasses.

As regards *A. donax* saccharification experiments performed with commercial enzyme cocktail Novozymes Cellic®, Ctec3 and Htec3, and rPoAbf, its evolved variant rPoAbf F435Y/Y446F and rCelStrep, no significant increase in sugar conversion was observed (Fig. 3a–c). However, it is worthy to note that supplementing rPoAbf F435Y/Y446F, the percentage of xylan and arabinan conversion increased, reaching the highest conversion (80 % for both sugars) after 6 days of hydrolysis. Comparing the data of conversion of *A. donax* pretreated by aqueous ammonia soaking (Marcolongo et al. 2014), similar xylan conversion was achieved by using the commercial mix in combination with rPoAbf and its evolved mutant after 72 h of hydrolysis. Even if the xylan hydrolysis described by Marcolongo et al. (2014) appeared to be faster than that reported in this work, it is worth to note that the amount of arabinofuranosidase enzymes in this manuscript (1.5 U/g expressed as units of purified enzyme, assayed against the substrate pNP-a-l-arabinofuranoside as previously described in Amore et al. 2012a, per gram of pretreated biomass) is lower than that (27.2 U/g) used in Marcolongo et al. (2014).

Also, in the case of corn stover, positive effects were observed in xylan and arabinan conversion when the enzymes tested in this work were added to the commercial enzyme cocktail Novozymes Cellic®, Ctec3 and Htec3. The 99 % xylan conversion was achieved in 24 h when rPoAbf wild type was supplemented to the commercial enzymes. The same xylan conversion was achieved using commercial preparation and commercial preparation in combination with rPoAbf F435Y/Y446F or rCelstrep or both enzymes after 6 days of hydrolysis. Interestingly, these data highlight that by adding rPoAbf wild type, the hydrolysis of corn stover seems to be faster than that with other preparations. These results could be due to the diverse degree of synergism among the enzymes due to different substrate proprieties as previously reported (Andersen et al. 2008; Van Dyk and Pletschke 2012).

All these data confirm the importance of using accessory enzymes like arabinofuranosidases to achieve high xylan conversion yield since they increase accessibility of xylan acting on side chain of hemicellulose and demonstrate the effectiveness of the arabinofuranosidase from the fungus *P. ostreatus* rPoAbf and, even more, of its evolved mutant rPoAbf F435Y/Y446F. It is worth to note that the use of the purified enzyme cocktail containing rPoAbf F435Y/Y446F allowed achieving higher or similar glucan, xylan and arabinan conversion after 72 h of hydrolysis (62, 63 and 80 %) than those achieved after the same time of hydrolysis with commercial enzymes Novozymes Cellic®, Ctec3 and Htec3 (44, 66 and 55 %).
